# Supplementary material for: Adaptive laboratory evolution reveals regulators involved in repressing biofilm development as key players in Bacillus subtilis root colonization
Source: mSystems. 2024 Jan 11;9(2):e00843-23. doi: 10.1128/msystems.00843-23 (PMC10878085; doi:10.1128/msystems.00843-23)
Supplement: Supplemental material — Supplemental figures and tables. [file msystems.00843-23-s0001.pdf]

Supplementary material

Table S1. Mutations identified in the genomes of isolates from the BRE on tomato roots.

Gene	Mutation		Lineage 1			Lineage 2							Lineage 3				Lineage 4				Lineage 5			Lineage 6		
			Cycle 7	Cycle 14	Cycle 21	Cycle 7	Cycle 14	Cycle 14	Cycle 14	Cycle 14	Cycle 21	Cycle 21	Cycle 7	Cycle 14	Cycle 14	Cycle 21	Cycle 7	Cycle 7	Cycle 14	Cycle 21	Cycle 7	Cycle 14	Cycle 21	Cycle 7	Cycle 14	Cycle 21
			1.a.C7	1.a.C14	1.a.C21	2.a.C7	2.a.C14	2.b.C14	2.c.C14	2.e.C14	2.a.C21	2.b.C21	3.a.C7	3.a.C14	3.b.C14	3.a.C21	4.a.C7	4.d.C7	4.a.C14	4.a.C21	5.a.C7	5.a.C14	5.a.C21	6.a.C7	6.a.C14	6.a.C21
ipk	c.241G>T	missense_variant												X												
B4U62_22510	c.523_*31del	stop_lost&inframe_deletion&splice_region_variant												X												
srfAC	c.3386C>A	missense_variant																								
yloN	c.157C>T	missense_variant						X																		
codY-flgB	n.1691261C>T	intergenic_region																								
yodI	c.104G>C	missense_variant							X																	
sinI-sinR	n.2552667G>A	intergenic_region																								
sinR	c.29G>T	missense_variant																			X	X	X			
	c.125C>T	missense_variant					X		X			X														
	c.296T>C	missense_variant											X	X	X	X										
	c.311G>T	missense_variant				X		X		X	X															
mccA	c.632G>T	missense_variant											X													
sdpA	c.77delA	frameshift_variant																								
epsC	c.1289T>G	missense_variant																								
ptkA	c.448G>T	stop_gained								X																
ywcC	c.250G>T	stop_gained																X	X							
	c.214_215delAA	frameshift_variant	X	X	X																					
	c.215delA	frameshift_variant																		X						
	c.11delA	frameshift_variant																						X	X	X
aldX	c.923_967delTCGAAAGAGGGGCGGAAGTGGTGTTCGGCGGCGTATTCGATGCCA	disruptive_inframe_deletion																		X						

Table S2. Mutations identified in the genomes of isolates from the BRPE on tomato root.

Gene	Mutation		Lineage 1					Lineage 2									Lineage 3					Lineage 4						Lineage 5				
			Cycle 7	Cycle 14	Cycle 14	Cycle 21	Cycle 21	Cycle 7	Cycle 14	Cycle 14	Cycle 14	Cycle 14	Cycle 14	Cycle 14	Cycle 14	Cycle 21	Cycle 7	Cycle 7	Cycle 14	Cycle 14	Cycle 21	Cycle 7	Cycle 14	Cycle 21	Cycle 21	Cycle 21	Cycle 21	Cycle 7	Cycle 14	Cycle 14	Cycle 21	Cycle 21
			1.a.C7	1.a.C14	1.c.C14	1.a.C21	1.b.C21	2.e.C7	2.a.C14	2.b.C14	2.c.C14	2.d.C14	2.d.C14	2.e.C14	2.f.C14	2.a.C21	3.a.C7	3.b.C7	3.a.C14	3.e.C14	3.a.C21	4.a.C7	4.b.C14	4.a.C21	4.b.C21	4.e.C21	4.f.C21	5.a.C7	5.a.C14	5.b.C14	5.a.C21	5.d.C21
ftsH	c.1269C>T	synonymous_variant												X																		
cshA	c.1357G>T	missense_variant								X																						
yesZ	c.1444G>T	stop_gained									X																					
slp-ykzW	n.1534070C>A	intergenic_region																						X								
ylzA	c.53_90delGGATGATTTCGATTGTCAGCCCGGAGTCTGCGCCAATC	frameshift_variant			X																											
flhA	c.926C>G	stop_gained						X																								
dxr	c.386C>T	missense_variant																	X		X											
pksM	c.1804C>T	missense_variant																		X												
pksR	c.5041T>C	missense_variant																				X	X	X	X	X	X					
ppsC	c.3511G>A	missense_variant														X																
ppsB	c.4737G>T	synonymous_variant														X																
ppsA-dacC	n.1998147delT	intergenic_region										X																				
yotL	c.107G>T	missense_variant		X																												
dxs	c.1246G>T	missense_variant																														X
	c.857C>G	missense_variant																													X	
sinI-sinR	n.2552661G>T	intergenic_region							X	X	X	X	X	X	X	X																
sinR	c.127T>G	missense_variant		X	X		X																									
	c.170C>T	missense_variant																											X		X	X
	c.296T>C	missense_variant																	X	X	X											
bscR-yrhH	n.2777799delT	intergenic_region																						X								
dhbE	c.776C>A	missense_variant				X																										
dhbC	c.695G>T	missense_variant	X																													
cssR	c.351G>C	synonymous_variant																X														
epsD	c.467_488delTTCAAAGGCCGGGCGGAAGGAC	frameshift_variant													X																	
epsC	c.141_142delTG	frameshift_variant											X																			
degU	c.436G>T	stop_gained																							X							
tagO	c.747A>G	missense_variant																									X					
gtaB	c.347G>T	missense_variant												X																		
	c.351_352delAC	frameshift_variant												X																		
	c.354_355insCT	frameshift_variant												X																		
	c.382G>T	missense_variant												X																		
	c.384A>T	synonymous_variant												X																		
	c.393T>A	synonymous_variant												X																		
B4U62_19240	c.26T>G	missense_variant												X																		
B4U62_19240	c.48T>C	synonymous_variant												X																		
ywcC	c.215delA	frameshift_variant																												X		
	c.11delA	frameshift_variant															X		X	X	X		X	X	X	X	X					
yxiO	c.685A>T	missense_variant																													X	

Table S3. Strains used in this study.

Strain	Genotype	Reference
NCIB 3610	WT/undomesticated	Lab stock[1]
PB389	3610 *amyE*::$P_{hyperspank}$-mKATE2	Richard Losick[2]
PB127	3610 *ywcC*::kan	YC295[3]
MP57	3610 *ywcC*::kan *amyE*::$P_{hyperspank}$-mKATE2	This study
PB18	3610 *sinR*::spc	Roberto Kolter[4]
MP63	3610 *sinR*::spc *amyE*::$P_{hyperspank}$-mKATE2	This study
BKK17220	168 *pksR*::kan	BGSC[5]
MP65	3610 *pksR*::kan	This study
MP66	3610 *pksR*::kan *amyE*::$P_{hyperspank}$-mKATE2	This study
BKE17220	168 pksR::erm	BGSC[5]
MP67	3610 *ywcC*::kan *pksR*::erm *amyE*::$P_{hyperspank}$-mKATE2	This study
MP60	3610 *ywcC*::kan sinR::spc	This study
MP68	3610 *ywcC::*kan *sinR*::spc *amyE*::$P_{hyperspank}$-mKATE2	This study
Pseudomonas fluorescens	WCS365	Roberto Kolter[6]
Pseudomonas fluorescens	WCS374	Cara Haney
Pseudomonas capeferrum	WCS358	Roberto Kolter
Pseudomonas protegens	Pf-5	Roberto Kolter
Pseudomonas protegens	CHA0	Cara Haney
Pseudomonas stutzeri	RCH2	Cara Haney

Antibiotics resistance abbreviations: Kanamycin (kan), spectinomycin (spc), erythromycin (erm)

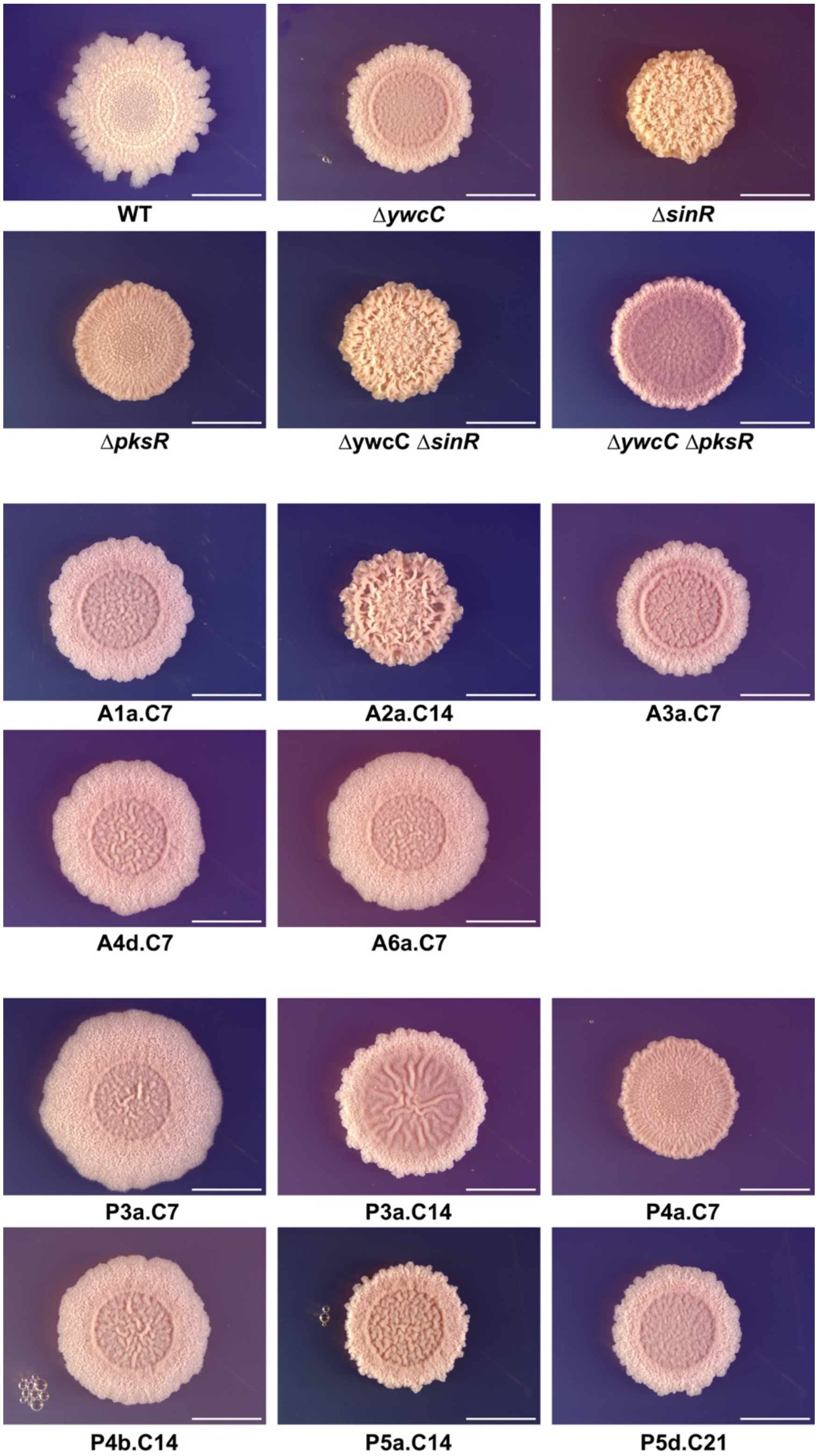

Fig S1. Morphotypes of deletion mutants and evolved isolates

Representative pictures of colony morphotypes of deletion mutants or evolved isolates grown on MS agar + 0.5% glutamate + 0.5% glycerol for 48 h. Scale bar is 5 mm for all images.

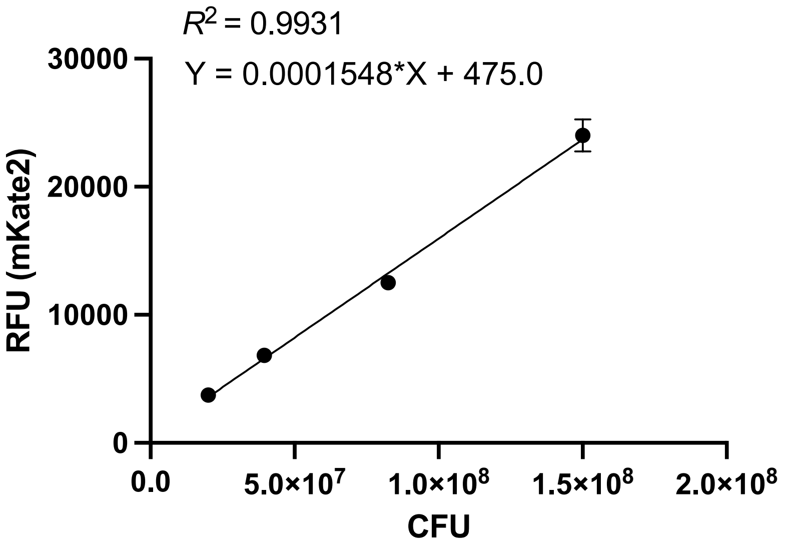

Fig S2. Relative fluorescence unit (RFU) is a quantitative tool which correlates with bacterial counts. RFU was measured using TECAN Spark monochromator-based (plate reader) at emission: 590 nm, excitation: 638 nm, and bacterial counts was followed by dilution and plating. Representative experiment of 3 replicates is presented.

Supplementary references

1. Branda, SS, González-Pastor, JE, Ben-Yehuda, S, Losick, R & Kolter, R. Fruiting body formation in Bacillus subtilis. *Proc Natl Acad Sci.* (2001) 98(20): 11621-11626.

2. Chen, Y., Cao, S., Chai, Y., Clardy, J., Kolter, R., Guo, J.-h. and Losick, R. A *Bacillus subtilis* sensor kinase involved in triggering biofilm formation on the roots of tomato plants. (2012) *Molecular Microbiology* 85: 418-430.

3. Chai Y, Kolter R, Losick R. Paralogous antirepressors acting on the master regulator for biofilm formation in Bacillus subtilis. *Molecular Microbiology* (2009) 74(4):876-87.

4. Kearns, D.B., Chu, F., Branda, S.S., Kolter, R. and Losick, R. A master regulator for biofilm formation by *Bacillus subtilis*. *Molecular Microbiology* (2005) 55: 739-749

5. Koo, B. *et al.* Libraries for *Bacillus subtilis*. *Cell Syst* **4**, (2017) 291-305.e7.

6. O'Toole GA, Kolter R. Initiation of biofilm formation in Pseudomonas fluorescens WCS365 proceeds via multiple, convergent signalling pathways: a genetic analysis. *Molecular Microbiology* (1998) May;28(3):449-61.
